# Supplementary material for: Human Evidence of Perfluorooctanoic Acid (PFOA) Exposure on Hepatic Disease: A Systematic Review and Meta-Analysis
Source: Int J Environ Res Public Health. 2022 Sep 8;19(18):11318. doi: 10.3390/ijerph191811318 (PMC9517074; doi:10.3390/ijerph191811318)
Supplement: Supplementary file 1 [file ijerph-19-11318-s001.zip › ijerph-1851634-supplementary.pdf]

Supplementary Table S1. PFOA database search strategy

Search term	
PubMed (search date: no date limit - 12/31/2021)	
1	“FC 143”[tiab] OR “fluorinated polymer”[tiab] OR “fluorinated polymers”[tiab] OR “pentadecafluoro-n-octanoic”[tiab] OR “perfluoro octanoate”[tiab] OR “perfluoro octanoic”[tiab] OR “perfluoro-n-octanoic”[tiab] OR “perfluorooctanoic acid”[tiab] OR “perfluorooctanoyl chloride”[tiab] OR "335-67-1"[tiab] OR "3825-26-1"[tiab] OR APFO[tiab] OR FC143[tiab] OR "fluorocarbon polymer"[tiab] OR "fluorocarbon polymers"[tiab] OR fluoropolymer*[tiab] OR fluorosurfactant*[tiab] OR fluorotelomer*[tiab] OR fluoro-telomer*[tiab] OR KPFOA[tiab] OR "ctanoic acid"[tiab] OR pentadecafluoro-[tiab] OR pentadecafluoroctanoic[tiab] OR "pentadecafluoro-n-octanoic acid"[tiab] OR pentadecafluorooctanoate*[tiab] OR pentadecafluorooctanoic[tiab] OR "perfluorinated C8"[tiab] OR "perfluorinated carboxylic"[tiab] OR "perfluorinated chemical*"[tiab] OR "perfluorinated compound*"[tiab] OR "perfluorinated octanoic"[tiab] OR perfluoroalkyl*[tiab] OR perfluorocaprylic[tiab] OR "perfluorocaprylic acid"[tiab] OR perfluorocarbon*[tiab] OR perfluorocarboxyl*[tiab] OR perfluorochemical*[tiab] OR perfluoroctanoate[tiab] OR perfluoroctanoic[tiab] OR "perfluoroctanoic acid"[tiab] OR "perfluoro-n-octanoic acid"[tiab] OR perfluorooctanoate[tiab] OR perfluorooctanoic[tiab] OR "perfluorooctanoic acid"[tiab] OR PFAA*[tiab] OR PFOA[tiab] OR "ammonium perfluorooctanoate"[tiab] OR "lithium perfluorooctanoate"[tiab] OR “hepatic toxicity”[tiab] OR liver[tiab]
2	“cohort studies”[tiab] OR “prospective studies”[tiab] OR “retrospective studies”[tiab] OR “epidemiologic studies”[tiab] OR “case-control studies”[tiab] OR “observational studies”[tiab] OR “follow-up studies”[tiab] OR “longitudinal studies”[tiab]
3	animals[mh] NOT human[mh]
4	#2 NOT #3
5	#1 AND #4
Search term	
EMBASE (search date: no date limit - 12/31/2021)	
1	“FC 143”:ab,ti OR “fluorinated polymer”:ab,ti OR “fluorinated polymers”:ab,ti OR “pentadecafluoro-n-octanoic”:ab,ti OR “perfluoro octanoate”:ab,ti OR “perfluoro octanoic”:ab,ti OR “perfluoro-n-octanoic”:ab,ti OR “perfluorooctanoic acid”:ab,ti OR “perfluorooctanoyl chloride”:ab,ti OR "335-67-1":ab,ti OR "3825-26-1":ab,ti OR APFO:ab,ti OR FC143:ab,ti OR "fluorocarbon polymer":ab,ti OR "fluorocarbon polymers":ab,ti OR fluoropolymer*:ab,ti OR fluorosurfactant*:ab,ti OR fluorotelomer*:ab,ti OR fluoro-telomer*:ab,ti OR KPFOA:ab,ti OR "octanoic acid":ab,ti OR pentadecafluoro-:ab,ti OR pentadecafluoroctanoic:ab,ti OR "pentadecafluoro-n-octanoic acid":ab,ti OR pentadecafluorooctanoate*:ab,ti OR pentadecafluorooctanoic:ab,ti OR "perfluorinated C8":ab,ti OR "perfluorinated carboxylic":ab,ti OR "perfluorinated chemical*":ab,ti OR "perfluorinated compound*":ab,ti OR "perfluorinated octanoic":ab,ti OR perfluoroalkyl*:ab,ti OR perfluorocaprylic:ab,ti OR "perfluorocaprylic acid":ab,ti OR perfluorocarbon*:ab,ti OR perfluorocarboxyl*:ab,ti OR perfluorochemical*:ab,ti OR perfluoroctanoate:ab,ti OR perfluoroctanoic:ab,ti OR "perfluoroctanoic acid":ab,ti OR "perfluoro-n-octanoic acid":ab,ti OR perfluorooctanoate:ab,ti OR perfluorooctanoic:ab,ti OR "perfluorooctanoic acid":ab,ti OR PFAA*:ab,ti OR PFOA:ab,ti OR "ammonium perfluorooctanoate":ab,ti OR "lithium perfluorooctanoate":ab,ti OR “hepatic toxicity”:ti,ab OR liver:ti,ab
2	“cohort studies”:ab,ti OR “prospective studies”:ab,ti OR “retrospective studies”:ab,ti OR “epidemiologic studies”:ab,ti OR “case-control studies”:ab,ti OR “observational studies”:ab,ti OR

	"follow-up studies":ab,ti OR "longitudinal studies":ab,ti
3	animals/exp NOT human/exp
4	#2 NOT #3
5	#1 AND #4
Search term	
Web of Science (search date: no date limit - 12/31/2021)	
1	TS="FC 143" OR TS="fluorinated polymer" OR TS="fluorinated polymers" OR TS="pentadecafluoro-n-octanoic" OR TS="perfluoro octanoate" OR TS="perfluoro octanoic" OR TS="perfluoro-n-octanoic" OR TS="perfluorooctanoic acid" OR TS="perfluorooctanoyl chloride" OR TS="335-67-1" OR TS="3825-26-1" OR TS=APFO OR TS=FC143 OR TS="fluorocarbon polymer" OR TS="fluorocarbon polymers" OR TS=fluoropolymer* OR TS=fluorosurfactant* OR TS=fluorotelomer* OR TS=fluoro-telomer* OR TS="KPFOA" OR TS="octanoic acid" OR TS=pentadecafluoro- OR TS=pentadecafluoroctanoic OR TS="pentadecafluoro-n-octanoic acid" OR TS=pentadecafluorooctanoate* OR TS=pentadecafluorooctanoic OR TS="perfluorinated C8" OR TS="perfluorinated carboxylic" OR TS="perfluorinated chemical*" OR TS="perfluorinated compound*" OR TS="perfluorinated octanoic" OR TS=perfluoroalkyl* OR TS=perfluorocaprylic OR TS="perfluorocaprylic acid" OR TS=perfluorocarbon* OR TS=perfluorocarboxyl* OR TS=perfluorochemical* OR TS=perfluoroctanoate OR TS=perfluoroctanoic OR TS="perfluoroctanoic acid" OR TS="perfluoro-n-octanoic acid" OR TS=perfluorooctanoate OR TS=perfluorooctanoic OR TS="perfluorooctanoic acid" OR TS=PFAA* OR TS=PFOA OR TS="ammonium perfluorooctanoate" OR TS="lithium perfluorooctanoate" OR TS="hepatic toxicity" OR TS=liver
2	TS="cohort studies" OR TS="prospective studies" OR TS="retrospective studies" OR TS="epidemiologic studies" OR TS="case-control studies" OR TS="observational studies" OR TS="follow-up studies" OR TS="longitudinal studies"
3	TS=animals NOT TS=human
4	#2 NOT #3
5	#1 AND #4

Supplementary Table S2. Title/abstract screening criteria for the literature searches

Variable	Inclusion criteria	Exclusion criteria
Populations	• Humans	• Ecological species
Exposures	• Exposure to a PFOA compound<br>• Exposure via oral, inhalation, dermal, intraperitoneal, or intravenous injection routes<br>• The level of exposure is measured using the trace in air, dust, drinking water, diet, gavage, or injection, or a biomarker of exposure	• Study population is not exposed to a PFOA compound<br>• Only exposed to a mixture
Outcomes	• Studies that include a measure of one or more health effect endpoints, including but not limited to, effects on all hepatic disease types<br>• Studies addressing risks to infants, children, pregnant women, occupational workers, the elderly, and any other susceptible or differentially exposed populations	• In which death was measured
Other		Not on topic, including:<br>• Abstract only, inadequately reported abstract, or no abstract and not considered further because study was not potentially relevant<br>• Ecosystem effects<br>• Studies describing the manufacture and use of PFOA compounds<br>• Not chemical specific (studies that do not involve testing of PFOA compound)<br>• Studies that describe measures of exposure to PFOA compounds without data on associated health effects<br>• Literature reviews, critical reviews, or cross-sectional study

Model	Effect size and 95% interval				Test of null (2-Tail)		Heterogeneity			
Model	Number Studies	Point estimate	Lower limit	Upper limit	Z-value	P-value	Q-value	df (Q)	P-value	I-squared
Fixed	2	1.169	1.089	1.256	4.296	0.000	0.249	1	0.618	0.000
Random	2	1,169	1.089	1.256	4.296	0.000				

Supplementary Figure S1. Overall average effect size

Study name	Odds ratio	Lower limit	Upper limit	Z-Value	p-Value	Relative weight
Darrow LA(2016)	1.172	1.091	1.259	4.325	0.000	98.69
Vieira VM(2013)	0.999	0.536	1.862	-0.003	0.997	1.31
	1.169	1.089	1.256	4.296	0.000	

Statistics for each study; Weight

Odds ratio and 95% CI

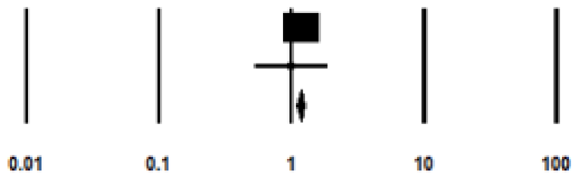

Supplementary Figure S2. A summary forest plot of effect size values for each study
